# 

**Published:** 1885-11

**Authors:** 


☞ INDUCEMENT.—In order to build up the Physicians' Mutual Benefit Association of Texas, and to induce our subscribers to remit their subscriptions NOW, we make this liberal proposition: All cash subscriptions from *new* subscribers during November and December will *pay for the* JOURNAL *to January*, 1887 —*fourteen months;* and, in addition, a *certificate of membership* in the Physicians' Mutual Benefit Association will be sent, *free of the membership fee* for 1885 (which is $1) to all who desire and request it. ☜

### IMPORTANT ANNOUNCMENT.

I take great pleasure in announcing that I have made arrangements with the well-known publishers, Dixon & Neatherly (Sam H. Dixon) to publish the BIOGRAPHY OF CONTEMPORARY PHYSICIANS OF TEXAS. These gentlemen are experienced book-publishers—having just issued the *Poets and Poetry of Texas*, and they will push the work rapidly to a completion, One of the firm will visit the physicians of the State and an opportunity will be given those who have not yet contributed their biographical data, to do so ; and those who have already subscribed but who have not yet sent in their biography are now urged to do so ere it be too late. There are thirty photographs on hand, of eminent, well-known practitioners and only a few more will be accepted. All communications relative to subscriptions, and the business of the publication should be addressed to the publishers. Biographical data from which a complete sketch is to be made, as well as photographs, should be sent to the undersigned. F. E. DANIEL, M. D., Editor.

### BIBLIOGRAPHY.

BOOKS RECEIVED.—We have received a number of valuable books, but, for want of space, we cannot do more than enumerate them at present. We will take an early opportunity, however, to review them at length. As to reprints and pamphlets, a mere list of them would occupy more room than we can spare. This thing of "reprints" is getting to be a nuisance.

*Index Catalogue Surgeon General's Office;* six large volumes. Courtesy of Dr. Billings.

*Ziemssen's Hand Book* of Therapeutics, etc., vol. 3. "The Therapeutics of the Respiratory Organs. Courtesy of Messrs. Wm. Wood & Co.

*The Science and Art of Midwifery;* Lusks. Courtesy of of Messrs. D. Appleton & Co., as also the following :

*Diseases of Children,* by Alfred Vogel.

*The Use of the Microscope*, Friedlander—Coe's translation.

*A Treatise on Nervous Diseases;* S. G. Webber.

*Physicians' Visiting List*, 1886; Lindsay & Blakistons'. Courtesy of Messrs. P. Blakiston & Co.

*Physicians' Day Book;* C. Henri Leenard, from author.

*Official Formulæ of Hospitals* ; courtesy of author, Dr. C. W. Taylor.

We have received, also, several new journals, notices of which we had prepared, but have not room for them ; also notices of several journal changes. We crave the indulgence of our confreres if we appear to be remiss in this respect.

